# Anatomical Correlation for Focused Assessment With Sonography in Trauma

**DOI:** 10.7759/cureus.37714

**Published:** 2023-04-17

**Authors:** Rylie Wackerly, Kathryn Thomas, Teresa Loomis, David Moeller, Mario Loomis

**Affiliations:** 1 Department of Clinical Anatomy, Sam Houston State University College of Osteopathic Medicine, Conroe, USA

**Keywords:** orientation fast exam, ultrasound anatomical correlation, cadaver correlation fast exam, fast exam, point of care ultrasound

## Abstract

When learning the Focused Assessment with Sonography in Trauma (FAST) exam, anatomical orientation can be difficult, especially in the subxiphoid and upper quadrant views. To facilitate understanding in these areas, a novel in-situ cadaver dissection was used to demonstrate anatomy related to the FAST exam. In situ, because the structures remained in normal positions with adjacent organs, layers, and spaces clearly visible from the point of view of the ultrasound probe. These views were then correlated with what was seen on the ultrasound screen. The right upper quadrant and subxiphoid anatomy were viewed in a mirror to match the ultrasound images, and the left upper quadrant was viewed directly from the examiner’s position, also matching the view on the ultrasound screen. The in-situ cadaver dissection was developed as a resource to correlate FAST exam ultrasound images in the upper quadrant and subxiphoid regions with related cadaver anatomy.

## Introduction

The classic hands-on approach to learning in medical residency education can be a beneficial type of near-peer teaching when the activity does not involve any risk or discomfort to the patient, but the nature of the Focused Assessment with Sonography in Trauma (FAST) exam is one in which time and proficiency are of the essence. Not only do novices take longer to complete the exam, but those with less experience tend to miss some important regions of interest [1]. Even if residents practice in non-trauma situations, it has been estimated that a resident would have to perform the FAST exam at least 50 times before becoming adequately proficient [2]. These 50 practice runs take time and while senior physicians can usually assess the urgency of a trauma situation and not allow learners to waste time when there is none to be wasted, unexpected scenarios can and do arise. It would be preferable, then, to shorten the learning curve by carrying out effective training outside of the trauma bay. In fact, an e-learning program focused on recognizing ultrasound images was found to accelerate first-year residents’ attainment of competency with the FAST exam such that they met and even surpassed that of second-year residents with more hands-on experience [3]. Since recognizing ultrasound images involves correlating that image with foundational anatomy, the use of a cadaver dissection specifically designed for that correlation should add yet another dimension to this preparatory learning. With this in mind, a novel cadaver dissection was designed to elucidate the in-situ anatomy related to the FAST exam.

## Technical report

A cadaver embalmed for medical education was used to develop a curricular module of anatomical correlations with the ultrasound FAST exam. Sam Houston State University Institutional Review Board ruled this research exempt (IRB# 2022-329). The cadaver utilized in this study was obtained from the McGovern Medical School Willed Body Program in Houston Texas. The cadaver was donated, de-identified, and does not fall under living human tissue.

**Figure 1:**
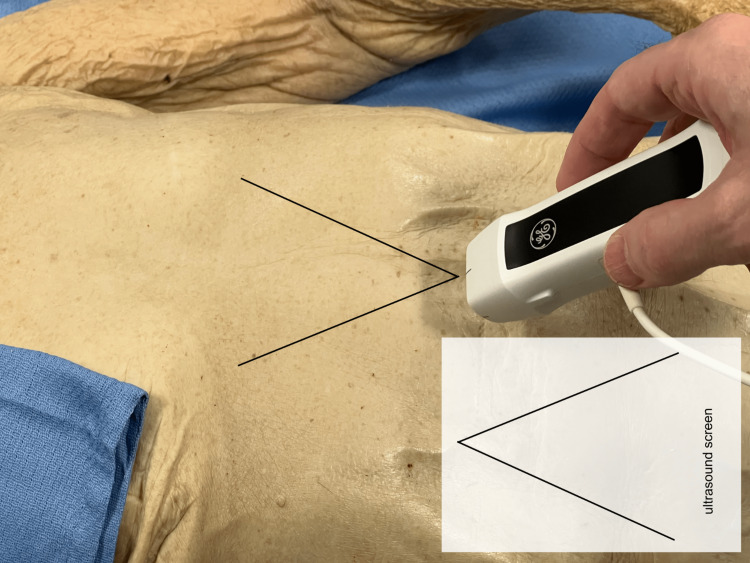
Subxiphoid probe An in-situ dissection of the chest was designed to demonstrate the view from the subxiphoid position of the ultrasound probe, which would appear as a reflection on the ultrasound screen.

The cadaver was used for other scholarly work and teaching demonstrations, sparing the abdomen and thoracic cavity, which were specially dissected for the ultrasound correlation. The perspectives were designed to mimic that of the ultrasound probe, beginning with the subxiphoid position. The reason why the image on the ultrasound screen would appear like a reflection of the gross structures was illustrated (Figure 1). 

**Figure 2:**
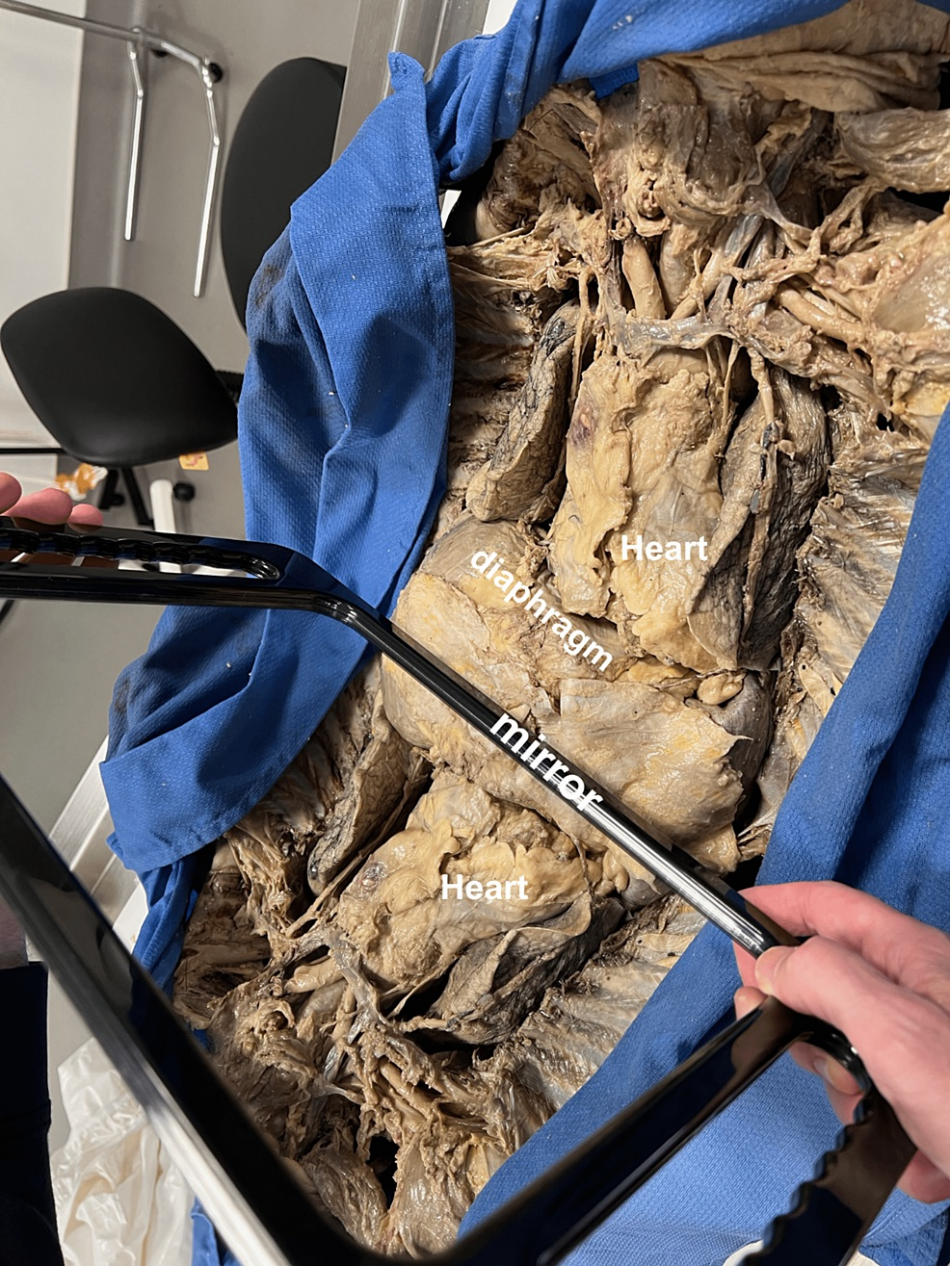
Subxiphoid mirror A mirror held in the position of the probe portrayed a reflection of the heart in its natural position, mimicking the view on the ultrasound screen.

The chest was opened with a median sternotomy, reflecting the rectus abdominis muscles inferiorly. The heart and lungs were kept in situ. The mediastinum was dissected only as needed to reveal the heart in its normal position. A mirror was used to correlate the gross anatomy with the image on the ultrasound screen (Figure 2).

**Figure 3:**
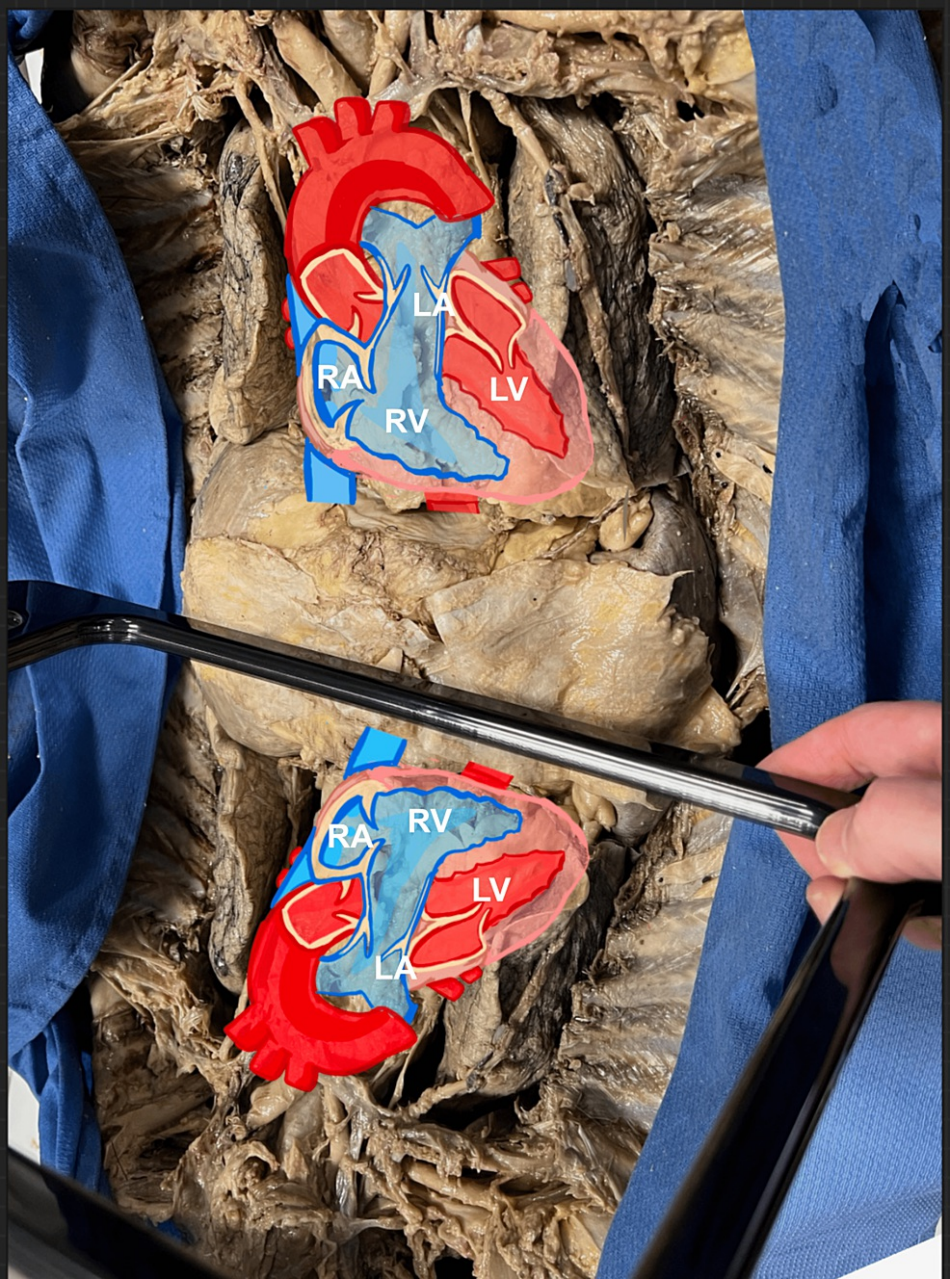
Subxiphoid mirror schematic Schematics over the heart and its reflection provided a visual impact of where each chamber was located on the ultrasound screen.

**Figure 4:**
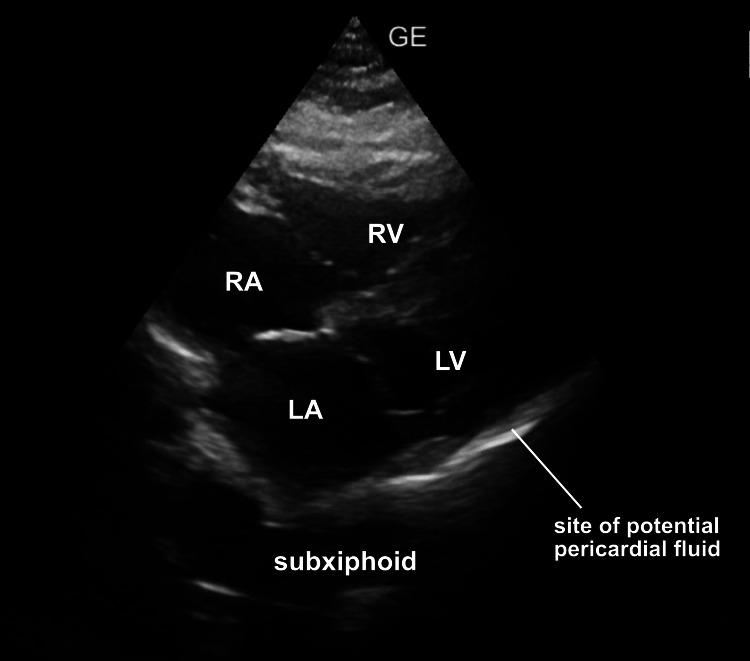
Subxiphoid ultrasound image The schematic correlated nicely with a typical subxiphoid view providing orientation to the chambers as well as the dependent area of the pericardial sac where one would look for abnormal fluid.

This image was later overlaid with schematics of the heart’s chambers (Figure 3), which correlated well with the ultrasound image at this site (Figure 4).

**Figure 5:**
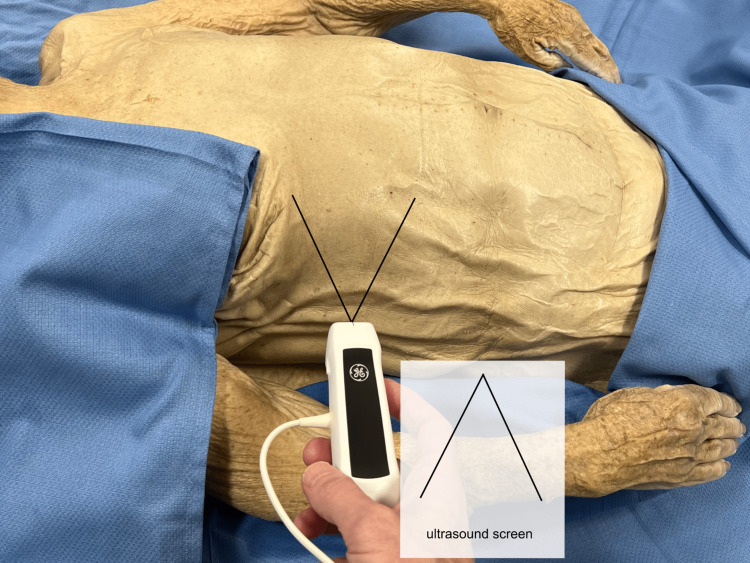
Right upper quadrant probe The perspective from the right upper quadrant would again need to be viewed as a reflection since the image on the ultrasound screen would be like a mirror image of the gross anatomy view.

The right upper quadrant was then addressed, illustrating again, the mirror-type relationship between the underlying structures and the image on the ultrasound screen (Figure 5). 

**Figure 6:**
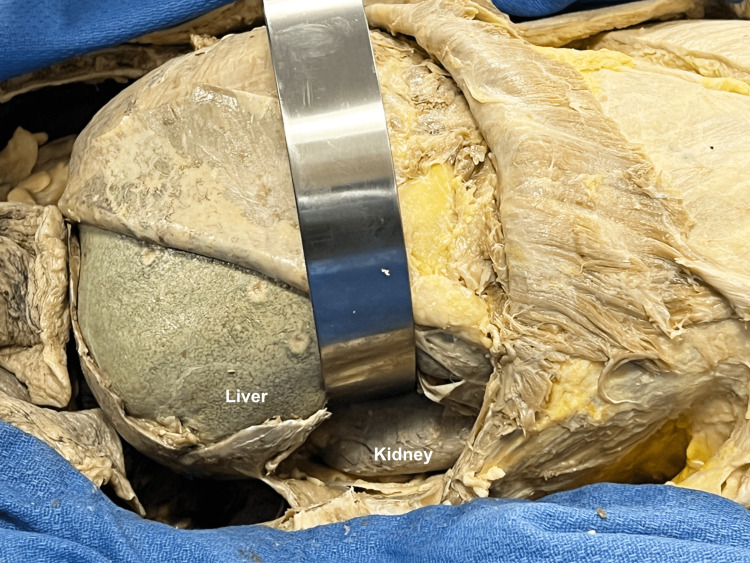
Right upper quadrant in situ The soft tissue and ribs were retracted to visualize the liver, kidney, diaphragm, and peritoneal layers in situ.

The external and internal oblique muscles were elevated off the ribs so that the ribs could be cut posteriorly and reflected downward. A block was used to prop up the right side of the cadaver so that the perspective would better match that of the probe, which is generally held down on the examining table. The diaphragm was incised and reflected medially and laterally, continuing the incision inferiorly through the parietal peritoneum down to the level of the hepatic flexure, and the retroperitoneum was entered to expose the kidney (Figure 6).

**Figure 7:**
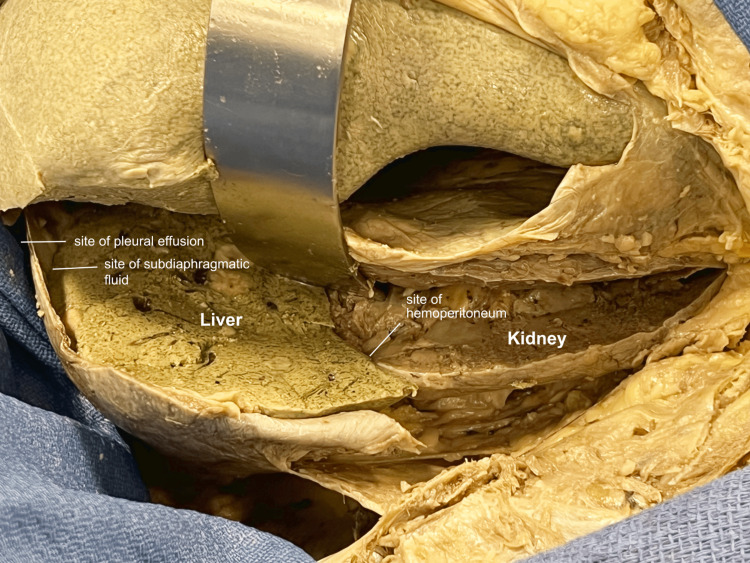
Right upper quadrant coronal section A coronal section of the region was performed to simulate the long-axis view of the ultrasound probe. The pleural, subdiaphragmatic, and hepatorenal spaces were noted.

A coronal-section incision was made to mimic the long-axis view of the ultrasound probe during the FAST exam. This involved transecting the liver and kidney in a coronal plane, including the adjacent diaphragm, pleural, and peritoneal layers (Figure 7).

**Figure 8:**
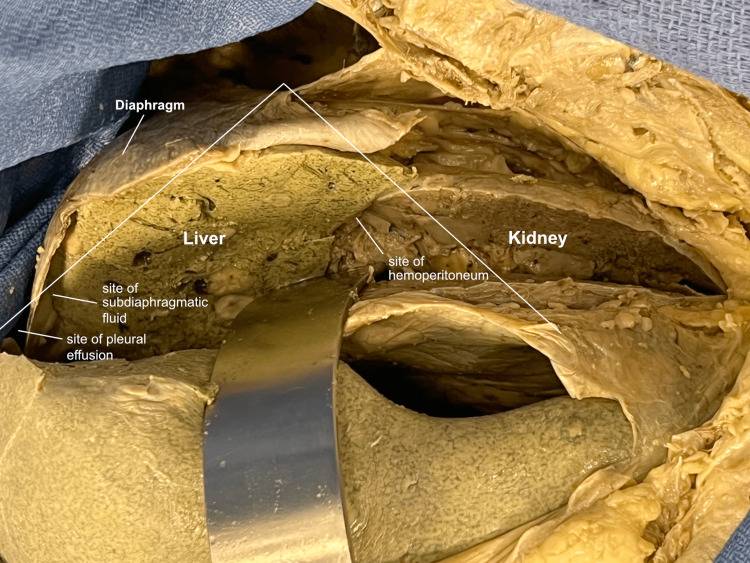
Right upper quadrant coronal-section mirror A mirror image of the structures was viewed to align with the image on the ultrasound screen, and areas of clinical relevance during the FAST exam were identified. FAST: Focused Assessment with Sonography in Trauma

This direct view was compared with a mirrored view, identifying key areas of interest identified during the FAST exam (Figure 8). 

**Figure 9:**
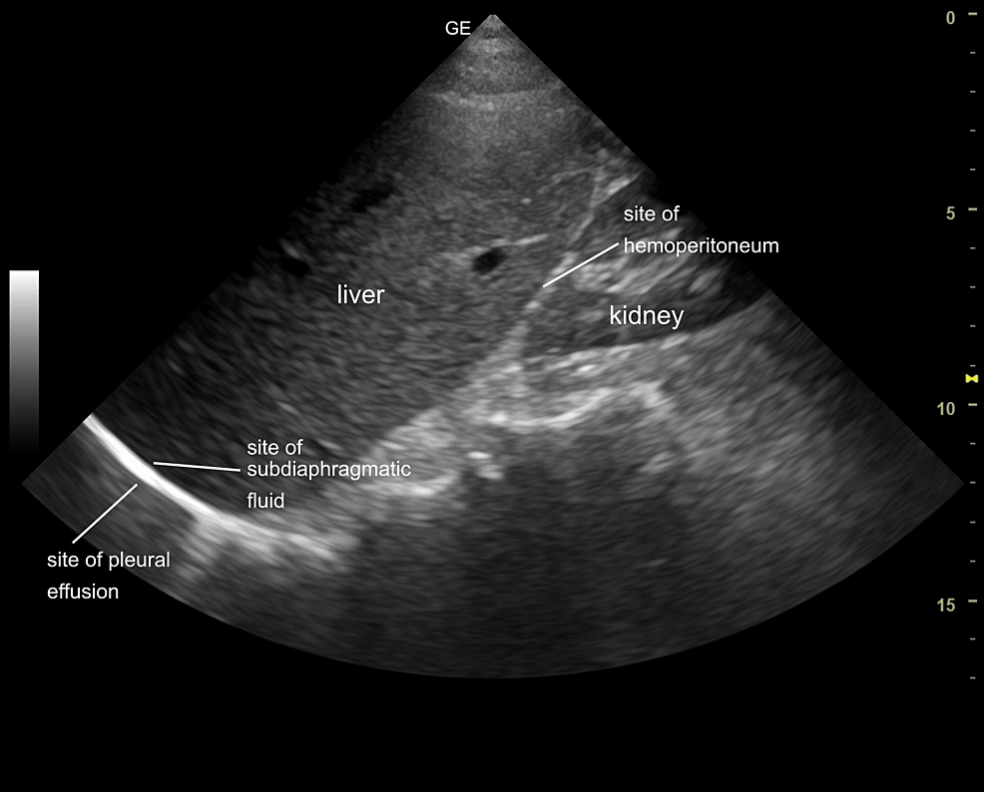
Right upper quadrant ultrasound image A typical ultrasound image was correlated with the gross anatomy and the sites of pleural, subdiaphragmatic, and hepatorenal fluid accumulation were identified.

The mirrored view correlated nicely with that typically seen on an ultrasound screen (Figure 9).

**Figure 10:**
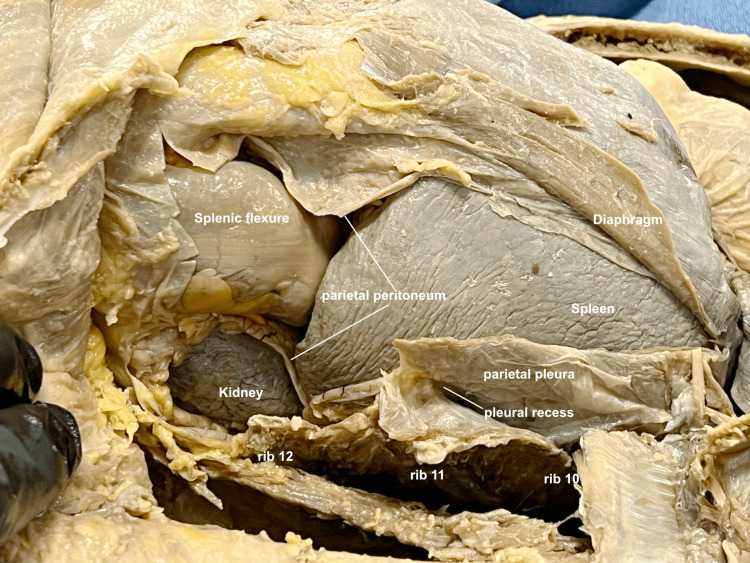
Left upper quadrant in situ The structures of the left upper quadrant were exposed in situ, noting the boundaries between pleural, peritoneal, and retroperitoneal spaces. (Note moderate splenomegaly is evident in this cadaver.)

The left upper quadrant was then addressed, reflecting the soft tissue and ribs in a similar fashion to the right upper quadrant. The diaphragm was incised and reflected, continuing the incision inferiorly through the parietal peritoneum to the level of the splenic flexure, and the retroperitoneum was entered to expose the kidney (Figure 10).

**Figure 11:**
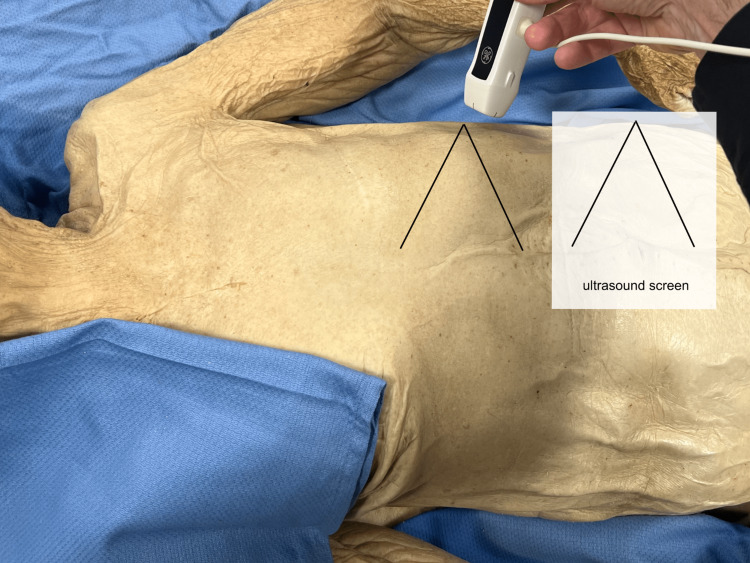
Left upper quadrant probe The view from an examiner’s perspective, since opposite the direction of the ultrasound probe, would not require a reflection for correlation.

It was illustrated how, in this instance, the examiner’s viewpoint would already be opposite that of the ultrasound probe, so the gross image would correlate directly with the image on the ultrasound screen (Figure 11).

**Figure 12:**
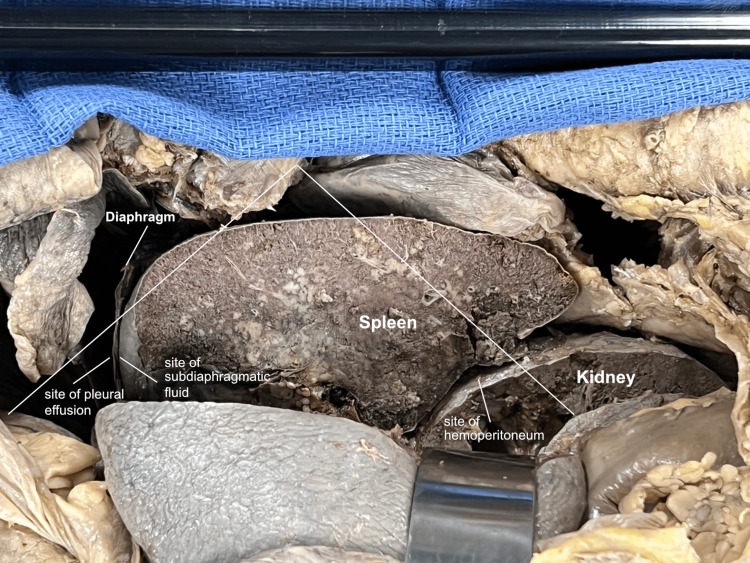
Left upper quadrant coronal section A coronal section of the left upper quadrant was performed to again simulate the long-axis view of the ultrasound probe. The view was like that of an examiner on the patient’s right side, leaning over the patient and looking down at the spleen from above. The pleural, subdiaphragmatic, and splenorenal spaces were noted.

**Figure 13:**
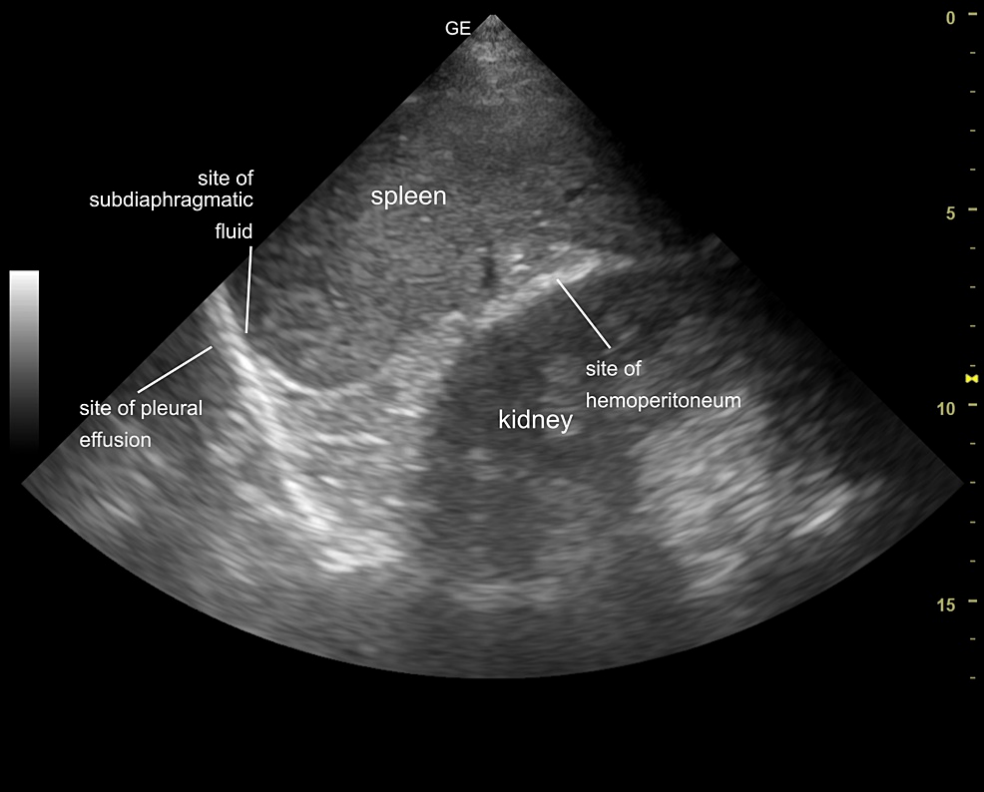
Left upper quadrant ultrasound image The gross view was correlated with the ultrasound image and the sites of potential fluid accumulation in the pleural, subdiaphragmatic, and peritoneal spaces were identified.

As in the right upper quadrant, a coronal-section incision was made to mimic the long-axis view of the ultrasound probe, transecting the spleen and kidney in a coronal plane, including the adjacent diaphragm, pleural, and peritoneal layers. The view looked down on the transected spleen and kidney from the perspective of an examiner standing on the patient’s right side. The key areas of interest sought during the FAST exam were identified on the gross view (Figure 12) and these were correlated with those on the ultrasound view (Figure 13).

## Discussion

Proficiency with the FAST exam involves not only dexterity and knowledge of machine settings, but also an ability to quickly orient oneself to the anatomy. This can be a challenge to those learning the exam as the ultrasound screen displays varying sections of structures and is oftentimes a mirror image as well. With repetition, pertinent images may be memorized and patterns recognized, but this type of learning, divorced from anatomical understanding, can be limiting. The perisplenic region can be a particularly confusing area in which to recognize pathology with its complex peritoneal spaces and folds [4]. The pericardial space, coined the “living” pericardial space by electrophysiologists for its nuanced anatomy, can be another challenge for the student of the FAST exam [5]. In such complex anatomical regions, accurate human perception and orientation are essential for the appropriate interpretation of medical imaging [6]. Ultrasound images by themselves do not present a complete anatomical picture, leaving gaps in understanding that can benefit from complimentary imaging studies [7]. Likewise, correlated anatomical dissections can help fill this gap in understanding as organs and surrounding spaces are appreciated in situ, helping the examiner envision and orient the anatomy even before the scan is started. The bladder and posterior cul-de-sac regions were not included in these dissections as we have found the cadaver views do not correlate well with live bladder ultrasounds. In cadavers, the bladder tends to be quite contracted or conversely, very dilated. In both cases, the normal relationship between the bladder and uterus or rectum is not well appreciated.

## Conclusions

A novel cadaver dissection was developed to correlate in-situ gross anatomy with the ultrasound images from the subxiphoid and upper quadrant views of the FAST exam to help learners quickly and accurately orient themselves in these areas. The gross anatomy images, viewed indirectly as mirror images in the subxiphoid and right upper quadrant views, and directly in the left upper quadrant view, correlated very well with the ultrasound images obtained during a FAST exam. It can take many repetitions for a student or resident to become competent at orienting themselves during an ultrasound exam. An area of future study would be to see if images such as these, of in-situ gross anatomy juxtaposed with related ultrasound images, prove to expedite the clinical learning process.
